# AGING AND FOREIGN POLICY: EXPERIENCING THE GLOBAL TRACK OF THE HEALTH AND AGING POLICY FELLOWSHIP

**DOI:** 10.1093/geroni/igad104.0023

**Published:** 2023-12-21

**Authors:** Jonathan Cohen

**Affiliations:** University of Southern California, Los Angeles, California, United States

## Abstract

The Health and Aging Policy Fellowship includes a Global Track that provides fellows with the opportunity to shape global policy or U.S. foreign policy for an aging society. The author, a global health expert and 2022-2023 fellow, sought a fellowship placement with a member of Congress with jurisdiction over U.S. global health policy. Although global health is a significant foreign policy priority for the U.S. government, U.S. global health programs do not include dedicated funding for activities designed to benefit older adults or help development partners adapt to population aging. The result is that US-funded programs on issues from infectious disease to health systems strengthening to disaster relief lack a focus on older adults, despite their many links to this growing population. To help address this policy gap, the author secured a fellowship placement with Senator Benjamin Cardin (D-MD), a senior member of the Senate Foreign Relations Committee (SFRC) and Chair of the SFRC Subcommittee overseeing the State Department, US Agency for International Development, and bilateral development assistance. With his track record of legislation and oversight on issues such as Medicare, health disparities, medical research, oral health, health systems strengthening, and retirement security, Senator Cardin is well positioned to help integrate the needs of older adults into U.S. development assistance. The author used his fellowship year to help advance the global health-related legislative priorities of Senator Cardin and his Health Team, bringing his expertise and perspective on aging to the policy table while gaining invaluable skills in the policy process.

